# Correlation between *Acinetobacter baumannii* Resistance and Hospital Use of Meropenem, Cefepime, and Ciprofloxacin: Time Series Analysis and Dynamic Regression Models

**DOI:** 10.3390/pathogens10040480

**Published:** 2021-04-15

**Authors:** Rania Kousovista, Christos Athanasiou, Konstantinos Liaskonis, Olga Ivopoulou, George Ismailos, Vangelis Karalis

**Affiliations:** 1 Department of Mathematics, University of Crete, Heraklion, 70013 Crete, Greece; rania.kousovista@yahoo.gr; 2 Pharmacy Department, General Military Hospital of Athens, 11525 Athens, Greece; 239chrisathan@gmail.com; 3 Department of Medical Biopathology, General Military Hospital of Athens, 11525 Athens, Greece; konanlia99@gmail.com (K.L.); olivopoulou@gmail.com (O.I.); 4 Experimental-Research Center ELPEN, ELPEN Pharmaceuticals, Pikermi, 19009 Attika, Greece; geosmile@elpen.gr; 5 Department of Pharmacy, School of Health Sciences, National and Kapodistrian University of Athens, 15784 Athens, Greece

**Keywords:** acinetobacter, antibiotic resistance, time series analysis, dynamic regression models, antimicrobial stewardship, meropenem, cefepime, ciprofloxacin

## Abstract

*Acinetobacter baumannii* is one of the most difficult-to-treat pathogens worldwide, due to developed resistance. The aim of this study was to evaluate the use of widely prescribed antimicrobials and the respective resistance rates of *A. baumannii,* and to explore the relationship between antimicrobial use and the emergence of *A. baumannii* resistance in a tertiary care hospital. Monthly data on *A. baumannii* susceptibility rates and antimicrobial use, between January 2014 and December 2017, were analyzed using time series analysis (Autoregressive Integrated Moving Average (ARIMA) models) and dynamic regression models. Temporal correlations between meropenem, cefepime, and ciprofloxacin use and the corresponding rates of *A. baumannii* resistance were documented. The results of ARIMA models showed statistically significant correlation between meropenem use and the detection rate of meropenem-resistant *A. baumannii* with a lag of two months (*p* = 0.024). A positive association, with one month lag, was identified between cefepime use and cefepime-resistant *A. baumannii* (*p* = 0.028), as well as between ciprofloxacin use and its resistance (*p* < 0.001). The dynamic regression models offered explanation of variance for the resistance rates (R^2^ > 0.60). The magnitude of the effect on resistance for each antimicrobial agent differed significantly.

## 1. Introduction

*Acinetobacter* species are Gram-negative bacteria associated with bacteremia and hospital-acquired pneumonia, including ventilator-associated pneumonia, surgical site infection, secondary meningitis, and urinary tract infections [1,2,3]. *Acinetobacter baumannii* has been reported as one of the most important and difficult-to-treat pathogens in the hospital setting [4,5]. Moreover, the number of *A. baumannii* infections is steadily increasing, while the optimal treatment of these infections has not yet been established [6]. The lack of new antimicrobial agents against *A. baumannii* and its ability to accumulate multiple antibiotic resistance genes has led to the current situation of multidrug-resistant (MDR) or extensively drug-resistant *Acinetobacter* isolates [7,8,9]. Multidrug resistance is considered to be non-susceptibility to at least one agent from three or more antibiotic classes that would otherwise serve as clinically effective treatments and has become a significant cause of increased morbidity and mortality in critically ill patients with severe sepsis [10,11].

Previous use of carbapenems, third- and fourth-generation cephalosporins, and fluoroquinolones are independent risk factors for acquisition of MDR-*Acinetobacter* [12,13]. Since carbapenems, especially meropenem, are frequently administered to treat *A. baumannii*, carbapenem-resistant *A. baumannii* (CRAB) strains have become a major therapeutic challenge in recent years [4,14,15]. CRAB is one of the critical priority pathogens on the World Health Organization priority list for antibiotic-resistant bacteria, which require effective drug development [16]. Cephalosporins could be another option for the treatment of *A. baumannii*, but cephalosporins are not usually considered suitable for the treatment of *A. baumannii* infections due to widespread high resistance rates [4]. High nosocomial *A. baumannii* resistance has also been observed for fluoroquinolones (e.g., ciprofloxacin) [17,18,19]. Recently, Butler et al. published a detailed study on the treatment options for MDR *A. baumannii* infections [20]. Despite the large number of studies on *A. baumannii* resistance, there are few data on the correlation between hospital antimicrobial use and resistance development, information that is critical for the development of an antimicrobial stewardship program (ASP) [21,22,23]. 

Numerous methods have been applied to investigate the above correlation; however, time series models and relevant analyzes, such as Autoregressive Integrated Moving Average models (ARIMA), dynamic regression models, are recognized as valuable tools for modeling antibiotic use-related resistance [24]. These methods offer the possibility of considering the influence of the timing of an intervention on this relationship, ensuring that the possible cause of antibiotic use precedes the effect of antibiotic resistance. These mathematical models also analyze the behavior of the dependent variable (antimicrobial resistance) as a function of its prior value, trends, and steep changes in the recent past.

Considering the above, the aim of this study was to use monthly hospital data to investigate the resistance rates of *A. baumannii* and the use of three broad-spectrum antimicrobial agents over a period of four years. In addition, the correlation of the above data was investigated by using time series analysis (ARIMA) and dynamic regression models.

## 2. Results

*A. baumannii* isolates were tested for susceptibility to all available antimicrobial agents and Clinical & Laboratory Standards Institute (CLSI) criteria were applied [25]. The evolution of mean yearly antimicrobial use expressed as DDD/100 patient days from January 2014 to December 2017 is shown in Table 1. It was observed that meropenem had the highest mean consumption of the antibiotics studied and its use increased significantly from 2014 to 2017. In addition, ciprofloxacin and the β-lactam combination piperacillin/tazobactam exhibited high levels of consumption per year, while imipenem use decreased gradually between 2014 and 2016 and eventually was practically annihilated in 2017. 

According to our data, a large number of *A. baumannii* strains were found to be multi-drug resistant, considering five classes of antibiotics that may be used against this pathogen (cephalosporins, fluoroquinolones, carbapenems, polymyxins and combinations of β-lactam/β-lactamase inhibitor). In particular, 15% of isolates were found to be resistant to all five classes, 42% were resistant to four classes, and 30% were resistant to three classes. Consequently, only 5.5% of the strains were sensitive to all the aforementioned antimicrobial classes. These findings are in accordance with previously reported data concerning the alarming resistance rates and resistance trends over time (1996–2017) of *A. baumannii* in Greek hospitals [27,28]. The distribution of isolates in terms of the biological sample and the hospital department is presented in Table 2. 

Time series analysis and transfer function models were applied to all antimicrobial agents. The only antimicrobials that presented a correlation between their use with their *A. baumannii* resistance pattern were meropenem, cefepime, and ciprofloxacin (Table 3, Table 4 and Table 5). No correlation between prior antimicrobial use and pathogen resistance rates was revealed for tigecycline, piperacillin/tazobactam, imipenem, and ceftazidime (Table A1).

### 2.1. Meropenem

**Figure 1:**
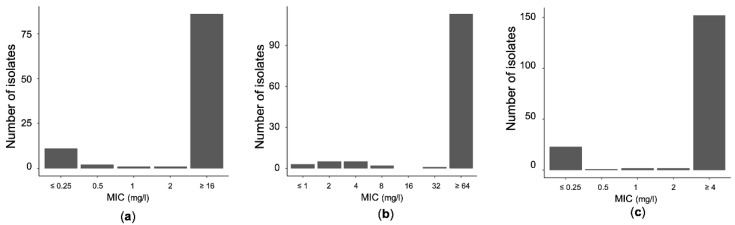
Distribution of meropenem (**a**), cefepime (**b**), and ciprofloxacin (**c**) minimum inhibitory concentrations (MICs) from *A. baumannii* blood isolates during the four-year period of the study.

Between January 2014 and December 2017, the observed mean detection rate of meropenem-resistant *A. baumannii* was 1.89 isolates per month; Figure 1a shows the meropenem Minimum Inhibitory Concentration (MIC) distribution of strains collected during the study period. 

**Figure 2:**
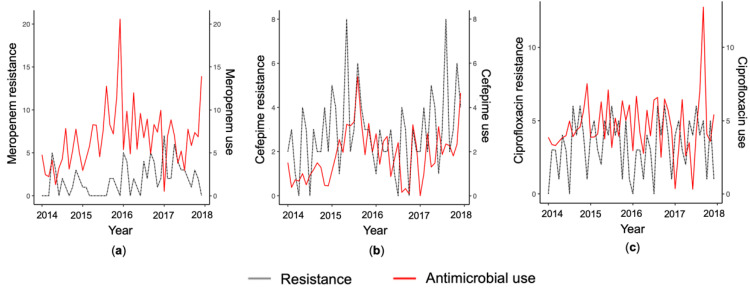
Smoothed monthly resistance detection rate of *A. baumannii* and hospital use of meropenem (**a**), cefepime (**b**), and ciprofloxacin (**c**). Usage is expressed in defined daily doses per 100 patient days.

The monthly mean use of meropenem was 6.74 Defined Daily Doses (DDDs) per 100 Patient Days (PDs). Smoothed data of meropenem use and meropenem-resistant *A. baumannii* are depicted in Figure 2a.

The ARIMA models and their corresponding parameters are shown in Table 3A,B for the meropenem-resistant *A. baumannii* and meropenem consumption series, respectively. Prior to these models, time series became stationary through differencing. Two significant autoregressive terms of orders (lags) of 1 and 2 months were identified for meropenem resistance. The coefficient of determination (R^2^) was 0.531 and the Akaike Information Criterion (AIC) was 186.73. In addition, three significant autoregressive terms of orders of 1, 2, and 3 months were identified for meropenem use; the R^2^ was 0.638 and the AIC was 242.91. Autocorrelation function (ACF) and partial autocorrelation function (PACF) plots of both model residuals confirmed that the series residuals corresponded to white noise.

To investigate a possible relationship between meropenem-resistant *A. baumannii* detection rate and meropenem use, we built a dynamic regression model via the linear transfer function (Table 3C). Examination of the cross-correlation function of the residuals of the two previous ARIMA models showed only one significant correlation with lag of 2 months. Therefore, two-month lags in the meropenem use time series were introduced in the transfer function model. The R^2^ of the transfer function model was 0.626 and the AIC was 166.25. The transfer function model could also be presented by the following equation:

R(t) = −0.564 R(t − 1) − 0.61 R(t − 2) + 0.13 U(t − 2) + e(t)(1)

where R is the detection rate of meropenem resistance observed at t months, U is the hospital meropenem use, and e(t) represents the residual error.

### 2.2. Cefepime

The mean monthly detection rate of cefepime-resistant *A. baumannii* was 2.37 isolates during the study period. The observed mean monthly cefepime consumption was 1.85 DDD/100 patient days. The distribution of MICs of cefepime is shown in Figure 1b. Smoothed data on cefepime use and cefepime-resistant *A. baumannii* are shown in Figure 2b.

Table 4 shows ARIMA and transfer function models for estimating the detection rate of cefepime resistance among *A. baumannii* isolates. The series were stationary in variance and mean after log transformation and simple differencing. Specifically, Table 4A shows an ARIMA model for cefepime resistance rate among *A. baumannii* that identified two significant autoregressive terms of order of 1 and 2 months. The AIC value was 100.56 and the R^2^ was 0.58. The ARIMA model of cefepime use included two significant autoregressive terms of order of 1 and 2 months (Table 4B). In this case, the AIC was 139.03 and the R^2^ was 0.619. ACF and PACF plots verified that the series residuals for the above models corresponded to white noise.

The influence of cefepime use in cefepime-resistant *A. baumannii* was analyzed using dynamic regression models, i.e., using the transfer function (Table 4C). A lag of one month was identified from the cross-correlation function between the residuals of the two previous ARIMA models. Accordingly, we introduced a one-month lag of cefepime use in the transfer function model and two significant autoregressive terms of order of 1 and 2 months of the residuals. The R^2^ was 0.66 and the AIC was 85.53.

### 2.3. Ciprofloxacin

The monthly mean of ciprofloxacin-resistant *A. baumannii* isolates was 3.16 isolates and the mean of ciprofloxacin use was 4.65 DDDs/100 patient days during the study period. Figure 1c presents the distribution of ciprofloxacin MICs and Figure 2c shows smoothed monthly series of ciprofloxacin use and ciprofloxacin-resistant *A. baumannii*.

ARIMA and transfer function models were built to estimate the detection rate of ciprofloxacin resistance among *A. baumannii* isolates (Table 5). 

The series were first differentiated to achieve stationarity. The ARIMA model of ciprofloxacin resistance rate of *A. baumannii* showed a significant first order moving average term (Table 5A). The AIC value was 99.38 and the R^2^ was 0.486. In addition, the identified model of the ciprofloxacin use series contained two significant autoregressive terms of order of 1 and 3 months (Table 5B). In this case, the AIC was 77.76 and the R_2_ was 0.55. Combining the above models, we developed a dynamic regression model of the ciprofloxacin-resistant *A. baumannii* detection rate and ciprofloxacin use by introducing a lag of 1 month in the ciprofloxacin use series (Table 5C). This lag was found by cross-correlating the residuals of the ARIMA models for ciprofloxacin use and resistance. The R_2_ was 0.617 and the AIC was 77.52.

**Figure 3:**
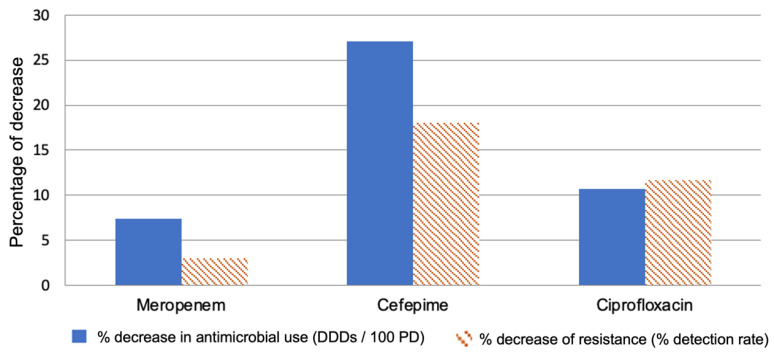
The predicted effect of a 0.5 Defined Daily Doses (DDD) per 100 patient days (PD) reduction for each antibiotic to the corresponding resistance.

Furthermore, in Figure 3 we can observe the possible impact of a 0.5 DDD/100 patient days on each of the antimicrobial agents. In the case of meropenem, the reduction is approximately 7.5% and could probably be achieved by 3% reduction in the detection rate. On the contrary, for ciprofloxacin the same reduction would mean a 10.75% decrease in use that could result in a significant resistance decrease (11.7%).

## 3. Discussion

In recent years, *A. baumannii* has developed resistance to virtually all known antibiotics [4,7]. Combination therapies of high-dose, long-term infusions of sulbactam, meropenem, ceftazidime/avibactam, and meropenem/varobactam with polymyxins, minocycline, aminoglycosides, and fosfomycin are being considered for the treatment of MDR *A. baumannii* infections [20,29,30]. Taconelli et al. proposed a priority list for research and development of new antibiotics for antibiotic-resistant bacteria and concluded that CRAB is a critical priority pathogen [16]. As a result, efforts must be made to maintain the susceptibility of this pathogen to currently available antimicrobials, especially ASPs. Although data on the correlation between antimicrobial use and resistance rates are required for effective work by ASP, we still have limited relevant data on *A. baumannii*.

In this study, ARIMA and transfer function models were identified to estimate the correlations between the detection rate of resistant *A. baumannii* isolates, previous resistance rates, and previous antimicrobial use. Our study provided significant results for three broad-spectrum antimicrobials, i.e., meropenem, ciprofloxacin, and cefepime. We identified an ARIMA model for the detection rate of meropenem-resistant *A. baumannii*; the rate was negatively related to the detection rate of the same resistance observed one and two months ago (Table 3A). The R^2^ was 0.531, meaning that 53% of the variance in this resistance series is anticipated by the model. Similarly, the ARIMA model for meropenem use showed a negative relationship with the same use series before one, two, and three months (Table 3B), where the R^2^ of the model was 0.638. Then, the influence of meropenem use in meropenem-resistant *A. baumannii* was analyzed using a dynamic regression model; according to it, the current rate of meropenem resistance was negatively related to resistance one and two months before and positively related to meropenem use two months before (Table 3C). An increase of 1 DDD/100 patient days for meropenem two months prior results in an increase of 0.13 in the meropenem resistance rate after accounting for meropenem use. In addition, ARIMA and transfer function models were estimated for the detection rate of cefepime resistance among *A. baumannii* isolates. These rates were negatively associated with the same rate one and two months ago (Table 4A), and the R^2^ of the model was 0.58. Cefepime use was also associated with the same use one and two months ago (Table 4B), and the R^2^ of the model was 0.619. The dynamic regression model implied that the current rate of cefepime resistance was negatively associated with resistance one and two months ago and positively associated with cefepime use one month ago (Table 4C). An increase of 1 DDD/100 patient days for cefepime two months prior results in an increase of 0.856 in the cefepime detection rate of resistant isolates after accounting for cefepime use. Finally, an ARIMA model for ciprofloxacin-resistant *A. baumannii* showed a negative association with the same series one month earlier (Table 5A). Ciprofloxacin use was negatively related to the same use one and three months ago and the coefficient of determination, R^2^, was 0.55 (Table 5B). The dynamic regression model showed that an increase in ciprofloxacin use by 1 DDD/100 patient days corresponded with an increase in the detection rate of ciprofloxacin-resistant isolates by 0.73 (Table 5C). These results are consistent with similar literature reports also indicating time delays between antimicrobial use and increased resistance [31,32,33]. For four other broad-spectrum antibiotics studied, i.e., tigecycline, piperacillin/tazobactam, imipenem, and ceftazidime, no correlation was found between antimicrobial use and corresponding resistance (Table A1).

It is worth noting that in all three models, the introduction of prior antimicrobial use significantly improved the models; the R^2^ increased to 0.6, meaning that more than 60% of the variance in the detection rate of resistant strains can be predicted by the described models. Figure 3 shows what a reduction of 0.5 DDD/100 PD would give for each of the antimicrobial agents studied; in relation to meropenem, this reduction, which is about 7.5%, could probably be achieved by an ASP but would give poor results, i.e., a 3% reduction in the detection rate. In contrast, the same reduction for ciprofloxacin would correspond to a 10.75% reduction in use, a still achievable target that could lead to a remarkable 11.7% reduction in resistance. This observation is an indication that ASPs should be planned based on local or regional epidemiological data, with pragmatic targets adapted to the results of the respective prediction models.

One of the limitations of this study is that the findings, on the association between antibiotic use and resistance, were examined on aggregate hospital data rather than at the individual patient level. In addition, molecular data were not available for the study period because genotypic testing is not routine in this hospital. Instead, data from phenotypic testing were used to inform antimicrobial treatment decisions. The benefits of molecular test information should be acknowledged, as this type of data would help in distinguishing between the different sources of resistance, e.g., horizontal genetic transfer or acquired resistance by mutation. The only limiting factor in the use of genotype data is its cost [34]. Despite the additional useful information that molecular data can provide, the phenotype data used in this study are adequate to fulfill the purpose of this study, which is to quantify the prior antimicrobial use and prior resistance rate on the observed resistance rate of *A. baumannii* [35]. It should also be recognized that mathematical models, despite their usefulness, have certain limitations, as they are based only on the mathematical properties of the series and not on the dynamics of infectious disease transmission [36]. Therefore, although the current results offered a reasonable level of reliability, future research may incorporate genotype data to gain better insight into the process of resistance of hospital-acquired infections.

Comparing our results with the literature, the complexity of the resistance phenomenon becomes clear; for example, with regard to meropenem, the results of this analysis are in agreement with previous studies, but no significant correlation of resistance was found with ceftazidime, while a strong positive correlation was found with the use of ciprofloxacin [23,37]. These observations contrast with the results of a recent study conducted in Serbia [21]. In addition, no effect of the rather limited imipenem use on resistance was found, which contrasts with the results of a Chinese study [38]. Such differences may be attributed to methodological reasons or may be due to the different epidemiological conditions in the hospital setting. Finally, another limitation of this study is that possible cross-correlations were not examined, such as the effect of ciprofloxacin use on meropenem resistance rates.

## 4. Materials and Methods

### 4.1. Clinical Setting

A retrospective study was performed from January 2014 to December 2017 in the General Military Hospital of Athens, a tertiary care hospital with medical and surgical wards as well as two Intensive Care Units (ICUs). This study research was approved by the hospital scientific committee. Informed consent was waived by the ethics review board. As to the hospital restriction policy, all prescriptions for broad spectrum antibiotics need composed endorsement by an infectious diseases specialist before administration; no adjustment in the limitation strategy or other significant change occurred during the investigation time frame.

### 4.2. Antibiotic Consumption

Monthly data of antimicrobial consumption were obtained for the study period from the hospital pharmacy database and were converted into defined daily doses (DDD). Antibiotic consumption was finally expressed as the number of defined daily doses/100 patient days according to the 2020 version of the ATC/DDD classification (World Health Organization Collaborating Centre for Drug Statistics Methodology, ATC/DDD index 2020) [26].

### 4.3. Microbiological Data

The results of susceptibility tests were obtained from the Clinical Microbiology Department of the hospital for clinical *A. baumannii* isolates for the study period. Interpretation of the results was performed according the CLSI criteria [25]. All clinical isolates of *A. baumannii* from every biological sample from all wards and ICUs were included in the analysis. The isolates with intermediate susceptibility were grouped with the resistant ones, forming the non-susceptible group. Detection rate of resistance was examined per month and was expressed as the number of multidrug-resistant *A. baumannii* isolates. Duplicate isolates were defined on the basis of the patient identity and the antibiotic phenotype.

### 4.4. Data Analysis

Prior to statistical analysis, any patients’ recognizable information was changed into anonymous information by the principal investigator of the study. A time series analysis of the monthly detection rate of *A. baumannii* resistance isolates and the monthly consumption of the respective broad-spectrum antimicrobials was performed. ARIMA models were used to analyze the temporal behavior of each variable in relation to its previous values, its trends, and any sudden changes. To identify an ARIMA model from the observed time series, it was first investigated whether the time series was stationary using the Augmented Dickey-Fuller test for Unit Roots. Then, the required assumptions of the Box and Jenkins methods were checked [39]. From the autocorrelation function and partial autocorrelation function, the appropriate order of the autoregressive and moving average terms of the model was selected. The parameters of the identified model were estimated using the maximum likelihood function or the unconditional least squares function. Goodness-of-fit criteria were estimated, such as the Akaike information criterion and the coefficient of determination, which corresponds to the percentage of variance in the observed time series explained by the model. Diagnostic checks, statistical significance of the parameters, and the ACF and PACF residuals of the model corresponded to white noise and were estimated to select the appropriate model. 

Once the basic ARIMA models were established, dynamic time series modeling techniques were used to assess the relationships between antimicrobial use series (DDD/100 patient days) and resistance series (detection of meropenem, cefepime, and ciprofloxacin *Acinetobacter* resistance isolates per 1000 patient days). In particular, the linear transfer function method proposed by Haugh (1976) was used to estimate these relationships [25]. The cross-correlation function between the residuals of these ARIMA models was calculated to identify the adequate lags in the antimicrobial use time series to be further introduced into the transfer function model. Cross-correlation of the resistance and use series was performed with lags of up to one year, and backward selection was applied to eliminate non-significant correlations. If no correlations were found, no lag order was selected. In addition, a diagnostic check, goodness-of-fit calculation, AIC, and R^2^ were implemented. The above techniques were analyzed using R software version 3.6.1 (the R project for statistical computing; http://www.r-project.org accessed on 15 April 2021).

## 5. Conclusions

In the present study, the association between the use of broad-spectrum antimicrobials (meropenem, cefepime, and ciprofloxacin) and resistant *A. baumannii* isolates in a tertiary hospital was investigated using time series analysis. Statistically significant associations were found between the use of these antimicrobials (meropenem, cefepime, ciprofloxacin) and corresponding resistance in *A. baumannii*. For each antimicrobial agent, a different pattern of correlation with resistance was revealed in terms of time lag and magnitude of effect. These results have important implications for strategies to contain resistance and highlight the need for antimicrobial stewardship programs to adjust their targets in accordance with locally developed predictive models.

## Figures and Tables

**Table 1:** Mean monthly antimicrobial consumption data^1^ estimated between January 2014 and December 2017.

Antimicrobial Agent	Year			
	2014	2015	2016	2017
Imipenem	1.29	1.10	0.41	0.02
Meropenem	4.28	8.49	7.77	6.42
Ceftazidime	0.51	0.31	0.62	0.48
Cefepime	0.88	2.75	1.69	2.08
Ciprofloxacin	4.41	4.89	4.86	4.44
Piperacillin/Tazobactam	3.59	3.88	4.45	3.84
Tigecyclin	0.91	1.72	2.08	2.69

^1^ Mean monthly use in Defined Daily Doses (DDD) [26] per 100 Patient Days (PD).

**Table 2:** Distribution of *A. baumannii* isolates gathered during the four-year study.

Per Specimen	Percent of Isolates	Per Department	Percent of Isolates
Blood	14.63%	Medical wards	52.44%
Urine	15.24%	Surgical wards	25%
Broncho-Alveolar Lavage	21.95%	Intensive Care Units	16.46%
Sputum	14.63%	Oncology/Hematological wards	4.88%
Trauma	11.59%	Mixed medical/surgical ward	1.22%
Other	21.95%		

**Table 3:** Autoregressive Integrated Moving Average (ARIMA) models for meropenem-resistant *A. baumannii* (**A**) and meropenem use (**B**). Dynamic regression model for the association between meropenem-resistant *A. baumannii* and hospital meropenem use (**C**).

Estimate	Model Parameter	Standard Error	*p*-Value
A. *A. baumannii* resistance			
ar1	−0.517	0.118	0.000
ar2	−0.578	0.114	<0.001
AIC	186.93		
R^2^	0.531		
B. Meropenem use (in DDD/100 PD)			
ar1	−0.831	0.122	<0.001
ar2	−0.637	0.144	<0.001
ar3	−0.569	0.117	<0.001
AIC	242.91		
R^2^	0.638		
C. Impact of meropenem use on *A. baumannii* resistance			
ar1	−0.564	0.126	<0.001
ar2	−0.610	0.124	<0.001
mer2	0.130	0.057	0.024
AIC	166.25		
R^2^	0.626		

Key: AIC, the estimated Akaike Information Criterion value for the model; ar1, autoregression term with a lag of one month of the ARIMA model; ar2, autoregressive component with lag equal to two months of the ARIMA model; ar3, autoregressive component with lag equal to three months of the ARIMA model; mer2, meropenem use of two months with lag time of two months; R2, the coefficient of determination of the model; DDD, Defined Daily Dose; PD, Patient days.

**Table 4:** ARIMA models for cefepime-resistant *A. baumannii* (**A**) and cefepime use (**B**). Dynamic regression model for the association between cefepime-resistant *A. baumannii* and hospital cefepime use (**C**).

Estimate	Model Parameter	Standard Error	*p*-Value
A. *A. baumannii* resistance			
ar1	−0.677	0.129	<0.001
ar2	−0.710	0.122	<0.001
AIC	100.56		
R^2^	0.580		
B. Cefepime use (in DDD/100 PD)			
ar1	−0.463	0.137	<0.001
ar2	−0.466	0.133	<0.001
AIC	139.03		
R^2^	0.619		
C. Impact of cefepime use on *A. baumannii* resistance			
ar1	−0.576	0.210	0.006
ar2	−0.559	0.222	0.011
cef1	0.865	0.395	0.028
AIC	85.53		
R^2^	0.660		

Key: AIC, the estimated Akaike Information Criterion value for the model; ar1, autoregression term with a lag of one month of the ARIMA model; ar2, autoregressive component with lag equal to two months of the ARIMA model; cef1, cefepime use of one month with lag time of one month; R^2^, the coefficient of determination of the model, DDD, Defined Daily Dose; PD, Patient days.

**Table 5:** ARIMA models for ciprofloxacin-resistant *A. baumannii* (**A**) and ciprofloxacin use (**B**). Dynamic regression model for the association between ciprofloxacin-resistant *A. baumannii* and hospital ciprofloxacin use (**C**).

Estimate	Model Parameter	Standard Error	*p*-Value
A. *A. baumannii* resistance			
ma1	−0.900	0.181	<0.001
AIC	99.38		
R^2^	0.486		
B. Ciprofloxacin use (in DDD/100 PD)			
ar1	−0.527	0.147	<0.001
ar3	−0.299	0.152	0.004
AIC	77.76		
R^2^	0.550		
C. Impact of ciprofloxacin use on *A. baumannii* resistance			
cip1	0.733	0.081	<0.001
AIC	77.52		
R^2^	0.617		

Key: AIC, the estimated Akaike Information Criterion value for the model; ar1, autoregression term with a lag of one month of the ARIMA model; ar3, autoregressive component with lag equal to three months of the ARIMA model; ma1, moving average component with lag equal to one month of the ARIMA model; cip1, ciprofloxacin use of one month with lag time of one month; R^2^, the coefficient of determination of the model; DDD, Defined Daily Dose; PD, Patient days.

## Data Availability

Data is contained within the article.

## Appendix A

**Table A1:** The association in univariate time series analysis between antimicrobial use and the corresponding antibiotic resistance of *A. baumannii*.

Antimicrobial Agent/Class	Order ^1^	*p*-Value ^2^	AIC ^3^	R^2^ 4
Imipenem	0	0.058	155.46	0.286
Ceftazidime	0	0.305	174.69	0.042
Piperacillin/Tazobactam	0	0.386	162.17	0.22
Tigecyclin	0	0.201	180.3	0.149

^1^ Delay before effect is observed (months).^2^ *p*-value for the association between antimicrobial use and *A. baumannii* resistance of the ARIMA model.^3^ Akaike information criterion.^4^ Coefficient of determination of the model.
